# Enriched Environment Decreases Cognitive Impairment in Elderly Rats With Prenatal Mobile Phone Exposure

**DOI:** 10.3389/fnagi.2020.00162

**Published:** 2020-06-04

**Authors:** Shanyan Hong, Honghong Huang, Meili Yang, Haining Wu, Lingxing Wang

**Affiliations:** Department of Neurology, The Second Affiliated Hospital of Fujian Medical University, Quanzhou, China

**Keywords:** prenatal, mobile phone exposure, cognitive impairment, enriched environment, offspring

## Abstract

Mobile phone use has rapidly increased worldwide, and pregnant women are passively or actively exposed to the associated electromagnetic radiation. Maternal cell phone exposure is related to behavioral difficulties in young offspring. However, whether prenatal mobile phone exposure can predispose the elderly offspring to cognitive impairment is unclear. The enriched environment (EE) has shown positive effects on cognition in an immature brain, but its impact on aging offspring after prenatal cell phone exposure is unknown. This study aimed to investigate whether prenatal exposure to mobile phone exerts long-term effects on cognition in elderly rat offspring and whether EE during adulthood can rescue cognitive impairment by altering the synaptic plasticity. Pregnant rats were subjected to prenatal short-term or long-term cell phone exposure and offspring rats were randomly assigned to standard or EE. Spatial learning and memory were investigated using Morris water maze (MWM) in elderly rat offspring. Hippocampal cellular morphology was assessed by hematoxylin-eosin staining and synaptic ultrastructure was evaluated with transmission electron microscopy. Expression of synaptophysin (SYN), postsynaptic density-95 (PSD-95), and brain-derived neurotrophic factor (BDNF) were detected by western blot. The results demonstrated that prenatal long-term but not short-term exposure to mobile phone lead to cognitive impairment, morphological changes in the hippocampal cells, reduced synaptic number, decreased SYN, PSD-95, and BDNF expression in elderly offspring, which were alleviated by postnatal EE housing. These findings suggest that prenatal long-term mobile phone exposure may pose life-long adverse effects on elderly offspring and impair cognition by disrupting the synaptic plasticity, which may be reversed by postnatal EE housing.

## Introduction

Mobile phone use has increased worldwide, especially in recent decades. Individuals including pregnant women are exposed to electromagnetic radiation emitted by mobile phones. It is possible for pregnant women to actively avoid electromagnetic radiation by reducing the use of mobile phones, however, passive exposure is omnipresent. A study has shown that high frequency maternal prenatal cell phone use is related to lower cognition in 5-year-old children (Sudan et al., 2018). Moreover, an association between maternal exposure to a cell phone during pregnancy and behavioral difficulties in children between the age of 7 and 11 years has also been reported (Divan et al., 2008). Studies were done in rodents also found a link between prenatal cell phone exposure and cognitive impairment in offspring (Aldad et al., 2012). However, all these studies are based on newborn, adolescent, or adult offspring, and the effect of maternal cell phone exposure on cognition in elderly offspring is unclear.

Enriched environment (EE), by providing novelty and extra space, is known to stimulate the brain using physical and social surroundings. A study has shown that EE has a beneficial effect on cognition and sensorimotor functions in normal animals (Garthe et al., 2016). It has also been demonstrated that EE helps the immature or adult brain in inducing a positive response after a neurological injury by trigging neurogenesis or increasing plasticity (Bondi et al., 2014; Bayat et al., 2015; Mering and Jolkkonen, 2015). Furthermore, EE can enhance plasticity and cognitive reserve in aged brains (Kempermann et al., 2002; Leal-Galicia et al., 2008) and even attenuate memory impairments due to significant synaptic protein loss in aged, socially isolated mice (Wang et al., 2018). All these results indicate a potential protective role of EE in elderly mice. However, whether EE can rescue neurological impairment in aged rat offspring after prenatal mobile phone exposure is unknown.

Therefore, in this study, we investigated the long-term impact of prenatal mobile phone exposure on cognition in elderly rat offspring. Furthermore, we explored whether EE can exert beneficial effects on cognitive function by modulating the synaptic plasticity in aged rats with prenatal mobile phone exposure.

## Materials and Methods

### Animals

The study was carried out following the Animal Care and Institutional Ethical Guidelines in China and was approved by the Ethics Committee of the Second Affiliated Hospital of Fujian Medical University. Sprague–Dawley rats were housed at room temperature (22 ± 1°C) with a 12 h light-dark cycle (7:00–19:00). Rats were allowed free access to food and water. Twelve week old female rats were mated with male rats in a 1:1 ratio. Vaginal smears were evaluated each morning for the presence of sperm. The day a vaginal plug or sperm was found was regarded as G0, and the female rat was defined as pregnant. Pregnant rats were randomly divided into no cell phone exposure (*n* = 5), short-term cell phone exposure (*n* = 5), and long-term cell phone exposure (*n* = 5) groups.

### Prenatal Cell Phone Exposure

Pregnant rats were housed, grouped, and subjected to cell phone exposure as previously described (Aldad et al., 2012). Briefly, rats in each group were placed in a cage with an 800–1900 MHz cell phone during the pregnancy (days 1–19 of pregnancy). The phone was positioned with the feeding bottle and the distance between rats and a cell phone was 7–40 cm. The cell phone in the short-term or long-term exposure group was in “active” call mode for 8 or 24 h, respectively, while cell phone in no exposure group was in standby mode. Two phones were used interchangeably to avoid any heat effects produced by the phones. At the end of day 19 of pregnancy, the cell phones were removed, and the pregnant rats were housed in separate cages until delivery.

### Housing Environment

At the age of 18 months, offspring rats in the prenatal cell phone exposure groups were randomly assigned to standard or EE, resulting in a total of five groups: control, prenatal short-term exposure + standard environment (PS + SE), prenatal short-term exposure + enriched environment (PS + EE), prenatal long-term exposure + standard environment (PL + SE), prenatal long-term exposure + enriched environment (PL + EE). Each group consisted of 12 rats, half male and half female. EE housing comprised of huge cages (60 × 45 × 76 cm) with plastic tunnels, running wheels, platforms, and colorful toys in different shapes and sizes. The objects were changed every 2 days. EE housing lasted for eight continuous weeks. At the same time, rats under standard environment were housed in normal cages, without any additional objects or toys.

### Morris Water Maze

Morris water maze (MWM) was conducted after eight continuous weeks of enriched or standard housing. The apparatus for MWM consists of a circular pool with a diameter of 160 cm and a height of 50 cm. The pool was divided into four equal quadrants and filled with water (22 ± 1°C). A platform (12 cm diameter) was hidden 1 cm below the surface of the water in the center of one of the quadrants. A video camera connected to the tracking software was positioned over the pool to record the movement. Rats were first trained for 5 days to find the platform. During a navigation trial, rats were put into the pool from any quadrant and allowed to move freely to find the platform, and the time was recorded. If a rat was unable to find the platform, it was guided to the platform and placed there for 15 s, and the time was recorded as 120 s. The trial was repeated four times per day for five continuous days and the time taken by a rat to locate the platform was regarded as escape latency. On each day, escape latency calculated in the four trials was averaged for statistical analysis. In the probing trial, the platform was removed. The number of platform crossings and the percentage time spent in the target quadrant by each rat was recorded.

### Tissue Collection and Histopathological Evaluation

Rats, under deep anesthesia, were transcardially perfused with saline, followed by 10% neutral paraformaldehyde. The brain was isolated and immersed in 10% formalin overnight. Subsequently, it was treated with a graded series of alcohol and xylene and was embedded in paraffin. The brain specimens were sliced into 5 μm thick sections using a microtome (Leica, Germany), which were stained with hematoxylin and eosin. The hippocampal cellular morphology was observed under a light microscope (Nikon, Japan).

Furthermore, the expression of synaptophysin (SYN) was detected by immunochemistry. The brain sections were dewaxed with xylene and rehydrated in graded ethanol. Endogenous peroxidase was blocked with 3% H_2_O_2_ after antigen retrieval. The sections were incubated overnight at 4°C with a diluted SYN rabbit polyclonal antibody (Biosynthesis Biotechnology Company, Beijing, China, 1:800). Next, the sections were washed in TBS and were incubated with biotinylated goat anti-rabbit secondary antibody (1:1,000; Biosynthesis Biotechnology Company, Beijing, China) for 30 min, which was visualized using diaminobenzidine (DAB) reagent. SYN-positive cells were observed under a light microscope. Furthermore, Image-Pro Plus 6.0 was used to measure the optical density (OD) average in the hippocampal CA1 region. A total of five sections of each rat were analyzed and the average OD of SYN was determined.

### Transmission Electron Microscopy

Rats were transcardially perfused with a mixture of 2.5% glutaraldehyde and 2% paraformaldehyde under anesthesia. Hippocampal samples were collected, immersed in 2% glutaraldehyde, and fixed with 1% osmic acid. After dehydration in graded series of alcohol, the samples were immersed in acetone and finally embedded in Epon 618 resin. Sections were cut and stained with uranyl acetate and lead citrate. H-7650 transmission electron microscope (Hitachi, Japan) was used to observe the ultrastructure of synapses. Five images were captured in a subregion per ultrathin section and the number of synapses was presented as an average number of synapses in each image at the same magnification.

### Western Blot

Hippocampi were quickly isolated from the brain and snap-frozen in liquid nitrogen. The hippocampal tissue was homogenized, and homogenates were centrifuged at 4°C. The concentration of total protein in the supernatant was detected using a BCA kit (Biosynthesis Biotechnology Company, Beijing, China). The supernatant was subsequently mixed with loading buffer and boiled for 3 min. An equal concentration of protein was separated with SDS-PAGE and transferred to PVDF membrane. The membrane was blocked with nonfat milk, followed by incubation with SYN (Biosynthesis Biotechnology Company, Beijing, China, 1:800), postsynaptic density-95 (PSD-95; Biosynthesis Biotechnology Company, Beijing, China, 1:800), brain-derived neurotrophic factor (BDNF; Biosynthesis Biotechnology Company, Beijing, China, 1:1,000) or β-actin (Biosynthesis Biotechnology Company, Beijing, China, 1:2,000) primary antibody overnight at 4°C. The membranes were incubated with horseradish peroxidase (HRP)-conjugated secondary antibody for 1 h at room temperature and visualized using ECL reagent. The consequent bands were then scanned (Epson, Japan), analyzed, and quantified with ImageJ.

### Statistical Analyses

SPSS 16.0 software was used for statistical analysis. Data are presented as mean ± SEM. A three-way repeated ANOVA was used to analyze escape latency (between-subject, prenatal exposure, and postnatal environment; within-subject, trial day) in the MWM test. The correlation of several platform crossings with SYN and PSD-95 expression was evaluated using Spearman’s correlation coefficient. The remaining data were analyzed by two-way ANOVA with prenatal exposure and the postnatal environment as the main fixed factors, followed by Tukey’s *post hoc* test. *P*-values of < 0.05 were considered statistically significant.

## Results

### Enriched Environment Attenuates Morphological Changes in the Hippocampus of Aged Offspring Following Prenatal Mobile Phone Exposure

**Figure 1:**
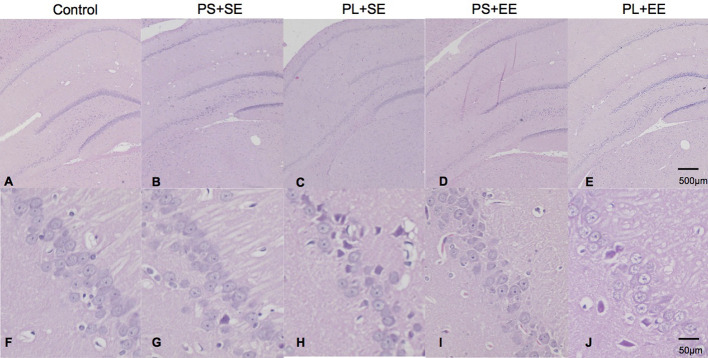
HE staining of hippocampus from aged rat offspring. Representative images of control **(A,F)**, prenatal short-term exposure + standard environment (PS + SE; **B,G**), prenatal long-term exposure + standard environment (PL + SE; **C,H**), prenatal short-term exposure + enriched environment (PS + EE; **D,I**), and prenatal long-term exposure + enriched environment (PL + EE; **E,J**) groups. Cells in the CA1 region of the PL + SE group were irregularly arranged with unclear nuclei as compared with the control group or PS + SE group. These morphological changes were alleviated in PL + EE group as compared with the PL + SE group (*n* = 4 per group).

Offspring from all the groups showed similar hippocampal architecture (Figures 1A–E). Microscopic examination exhibited round cells arranged regularly with clear and vesicular nuclei in the hippocampus of the control, PS + SE, and PS + EE groups (Figures 1F,G,I). However, cells in the CA1 region of the PL + SE group lacked normal arrangement, and a small subset of cells was irregular in shape with disrupted nuclei surrounded by vacuolated area (Figure 1H), and cellular changes were not obvious in CA2, CA3, or DG. Moreover, these morphological changes were rescued in the PL + EE group (Figure 1J) relative to the PL + SE group.

### Enriched Environment Strengthens Cognitive Function in Aged Rats With Prenatal Mobile Phone Exposure

**Figure 2:**
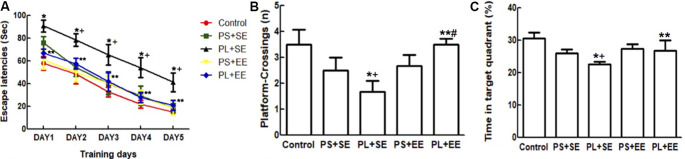
Effect of EE on morris water maze (MWM) performance in aged rat offspring after prenatal mobile phone exposure. **(A)** Escape latency during five successive days of navigation trial. **(B)** Number of crossings of the site in the probing trial where the platform had been placed. **(C)** Time spent in the target quadrant. Data are expressed as mean ± SEM, *n* = 12 per group. *p < 0.05, PL + SE group vs. control group; ^+^*p* < 0.05, PL + SE group vs. PS + SE group; **p < 0.05, PL + EE group vs. PL + SE group; ^#^*p* < 0.05, PL + EE group vs. PS + EE group.

A repeated ANOVA showed significant effects of prenatal phone × postnatal environment (*F*_(1,15)_ = 6.125, *P* = 0.016) on escape latency; however, no such effect was observed with prenatal phone exposure × postnatal environment × trial day, prenatal phone exposure × day, or postnatal environment × day. Moreover, significant effects were observed for day (*F*_(1,55)_ = 252.205, *P* = 0.000), prenatal phone exposure (*F*_(2,55)_ = 14.433, *P* = 0.000), and postnatal environment (*F*_(1,15)_ = 12.520, *P* = 0.001). Escape latency improved in a 5-day navigation trial for all groups; latency on day 1 was significantly longer than subsequent days (*p* < 0.05). Compared with the control group, latency in the PL + SE group was significantly increased, but not in the PS + SE group (*p* < 0.05, Figure 2A). Escape latency was significantly reduced in PL + EE group as compared with the PL + SE group (*p* < 0.05), however, no difference was found between PS + EE and PS + SE groups.

In the probing trial (Figures 2B,C), a two-way ANOVA analysis indicated that there was significant main effect of prenatal exposure (number, *F*_(2,55)_ = 9.966, *P* = 0.000; time, *F*_(2,55)_ = 14.102, *P* = 0.000) and postnatal environment (number, *F*_(1,55)_ = 17.483, *P* = 0.000; time, *F*_(1,55)_ = 7.509, *P* = 0.008), as well as a significant interaction between these two factors (number, *F*_(1,55)_ = 14.691, *P* = 0.000; time, *F*_(1,55)_ = 4.2790, *P* = 0.043) on the number of platform crossings and percentage time spent in the target quadrant. The PL offspring demonstrated fewer entries on the platform and spent less time in the target quadrant than the offspring of PS or control animals housed in the same standard environment (PL + SE vs. control, *p* < 0.05; PL + SE vs. PS + SE, *p* < 0.05); however, no difference was found between the PS + SE and control groups. There was a significant difference between the PL + EE and PS + EE groups (*p* < 0.05) for platform crossing numbers but not for the percentage time spent in the target quadrant. Moreover, the housing of the PL offspring in EE reversed the decrease in the number of platform crossings and percentage time spent in the target quadrant (PL + EE vs. PL + SE, *p* < 0.05), but no difference was observed in the PS + EE group as compared with the PS + SE group.

### Enriched Environment Alleviates Changes in Synaptic Ultrastructure and Synapse-Associated Proteins Induced by Prenatal Mobile Phone Exposure

**Figure 3:**
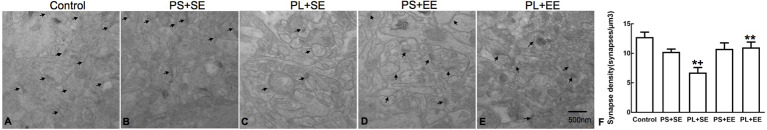
Transmission electron microscopy of synaptic ultrastructure. **(A–E)** Representative images of synaptic ultrastructure from hippocampus of different groups. **(F)** Summary data of synaptic numbers in the hippocampus of elderly rat offspring. Arrows indicate the number of synapses **(A–E)**. Data are expressed as mean ± SEM, *n* = 4 per group, *p < 0.05, PL + SE group vs. control group; ^+^*p* < 0.05, PL + SE group vs. PS + SE group; **p < 0.05, PL + EE group vs. PL + SE group.

The number of hippocampal synapses is shown in Figure 3. A two-way ANOVA revealed a significant main effect of prenatal phone exposure (*F*_(2,15)_ = 10.243, *P* = 0.002) and postnatal environment (*F*_(1,15)_ = 7.871, *P* = 0.013), as well as significant interaction between these two factors (*F*_(1,15)_ = 4.906, *P* = 0.033). *Post hoc* analysis showed that in standard housing environment, the PL group had a significantly low synaptic number in aged offspring as compared with the PS or control groups (PL + SE vs. control, *p* < 0.05; PL + SE vs. PS + SE, *p* < 0.05), whereas no difference was found between the PS + SE and control groups or between the PL + EE and PS + EE groups. Moreover, EE significantly prevented the pl-associated decrease in synaptic number (PL + EE vs. PL + SE, *p* < 0.05); however, this effect was not significantly different between the PS + EE and PS + SE groups.

**Figure 4:**
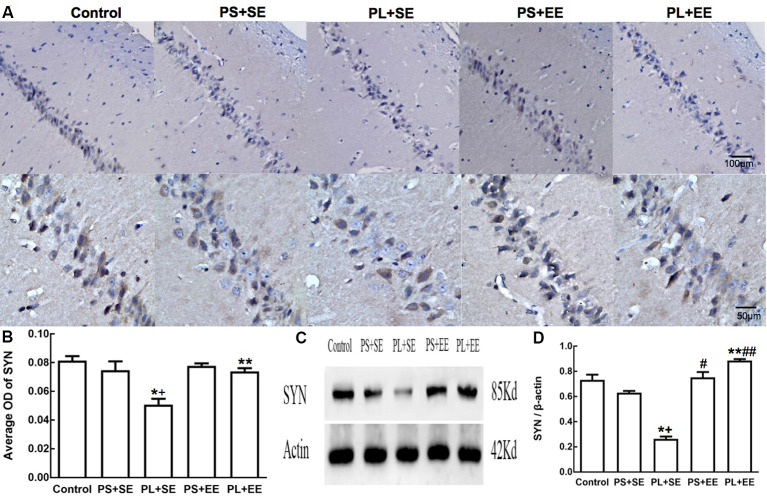
Expression of synaptophysin (SYN) in hippocampus of elderly rat offspring. **(A)** Representative photomicrographs of SYN expression by immunohistochemical staining. **(B)** Average optical density (OD) of SYN expression by immunohistochemistry. **(C,D)** Western blot and densitometric quantification of SYN expression (*n* = 4 per group). *p < 0.05, PL + SE group vs. control group; ^+^*p* < 0.05, PL + SE group vs. PS + SE group; **p < 0.05, PL + EE group vs. PL + SE group; ^#^*p* < 0.05, PS + EE group vs. PS + SE group; ^##^*p* < 0.05, PL + EE group vs. PS + EE group.

Immunohistochemical expression of SYN was observed as brownish-yellow granules in the hippocampal cells. Weak staining was observed in the hippocampal cells of PL + SE offspring as compared with the control or PS + SE offspring, and the staining was enhanced in the PL + EE group (Figure 4A). The average OD of SYN is shown in Figure 4B. A two-way ANOVA indicated a significant main effect of prenatal phone exposure (*F*_(2,15)_ = 60.055, *P* = 0.000), postnatal environment (*F*_(1,15)_ = 28.844, *P* = 0.000) and significant interaction of these two factors (*F*_(1,15)_ = 28.844, *P* = 0.000). *Post hoc* analysis revealed that at the standard environment, PL group had decreased average OD of SYN compared with control or PS group (PL + SE vs. control, *p* < 0.05; PL + SE vs. PS + SE, *p* < 0.05), however, there was no significant difference between PS + SE and control groups or between PL + EE and PS + EE groups. Furthermore, EE increased the averaged OD of SYN in the PL group when compared with standard housing (PL + EE vs. PL + SE, *p* < 0.05). No significant difference was found between PS + EE and PS + SE groups.

**Figure 5:**
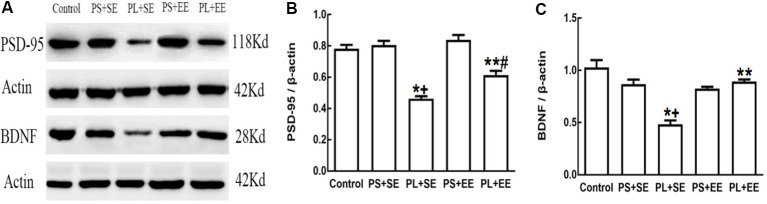
Expression of postsynaptic density-95 (PSD-95) and brain-derived neurotrophic factor (BDNF) in elderly rat offspring. **(A)** Representative immunoblots of PSD-95 and BDNF. **(B,C)** Quantification of PSD-95 and BDNF expression (*n* = 4 per group). *p < 0.05, PL + SE group vs. control group; ^+^*p* < 0.05, PL + SE group vs. PS + SE group; **p < 0.05, PL + EE group vs. PL + SE group; ^#^*p* < 0.05, PL + EE group vs. PS + EE group.

On quantitative assessment of western blot monograms (Figures 4C,D, 5A,B), analysis of variance showed a significant main effect of prenatal phone exposure on SYN and PSD-95 expression (SYN: *F*_(2,15)_ = 40.473, *P* = 0.000; PSD-95: *F*_(2,15)_ = 73.000, *P* = 0.000), a significant main effect of postnatal environment on SYN and PSD-95 expression (SYN: *F*_(1,15)_ = 164.128, *P* = 0.000; PSD-95: *F*_(1,15)_ = 13.470, *P* = 0.002), and a significant interaction between both the factors on SYN and PSD-95 expression (SYN: *F*_(1,15)_ = 73.928, *P* = 0.000; PSD-95: *F*_(1,15)_ = 5.290, *P* = 0.036). *Post hoc* analysis revealed that PL significantly reduced SYN and PSD-95 expression than PS or no prenatal phone exposure in aged offspring who were raised in the standard environment (PL + SE vs. control, *p* < 0.05; PL + SE vs. PS + SE, *p* < 0.05); however, no significant reduction was observed in the PS + SE group as compared with the control group. Also, SYN and PSD-95 expression were significantly different between the PL + EE and PS + EE groups (*p* < 0.05). Furthermore, EE significantly increased SYN and PSD-95 expression in aged offspring after PL exposure (PL + EE vs. PL + SE, *p* < 0.05); in contrast, expression of only SYN, but not PSD-95, was significantly up-regulated in the PS + EE group as compared with the PS + SE group (*p* < 0.05).

### Enriched Environment Increases Expression of BDNF in the Hippocampus of Aged Offspring With Prenatal Long-Term Mobile Phone Exposure

As shown in Figures 5A,C, there was a significant main effect of prenatal phone exposure (*F*_(2,15)_ = 29.358, *P* = 0.000) and postnatal environment (*F*_(1,15)_ = 18.350, *P* = 0.001) on BDNF expression, and a significant interaction was observed between these factors (*F*_(1,15)_ = 27.820, *P* = 0.000). *Post hoc* analysis indicated that PL significantly downregulated BDNF expression in the offspring at the standard environment as compared with control or PS-exposed offspring (PL + SE vs. control, *p* < 0.05; PL + SE vs. PS + SE, *p* < 0.05), whereas no significant difference was observed between the PS + SE and control groups or between the PL + EE and PS + EE group. EE significantly reversed PL-induced reduction in BDNF expression (PL + EE vs. PL + SE, *p* < 0.05), but no significant difference in BDNF expression was found between the PS + EE and PS + SE groups.

### Correlation of Cognitive Function With SYN and PSD-95 Expression

The number of platform crossings is an index of memory (Wang et al., 2019). Platform crossing number was found to be positively correlated with SYN (*r* = 0.71, *p* < 0.05) and PSD-95 (*r* = 0.65, *p* < 0.05) expression.

## Discussion

Epidemiological and experimental studies have shown that an insult or stimuli during the perinatal period might exert long-term effects on tissue structure and function in later life, which is also known as fetal origins of adult disease hypothesis (Langley-Evans, 2006). Under this hypothesis, our study showed that prenatal long-term mobile phone exposure can cause cognitive impairment, changes in hippocampal morphology and synaptic ultrastructure, decreased expression of synaptic proteins, and BDNF in the elderly offspring. Moreover, EE housing significantly improved cognitive function and inhibited adverse biochemical changes induced by prenatal long-term exposure to mobile phones in elderly rat offspring.

MWM is a classic and powerful technique to evaluate memory and spatial learning (Singh et al., 2016). An improvement in performance was observed in the rat offspring of all the groups during training. However, offspring in prenatal long-term mobile phone exposure group exhibited longer escape latency, fewer numbers of platform crossing, and lesser time spent in the target quadrant as compared to the control group, which indicates impaired spatial learning and memory upon long-term mobile phone exposure. In agreement with our results, studies have demonstrated that maternal exposure to radiofrequency radiation from cellular telephone affects learning acquisition and memory retention in rodent offspring (Aldad et al., 2012; Razavinasab et al., 2016). Investigations in humans have also shown that lower cognition score in children is related to maternal cell phone use during pregnancy. Nonetheless, negative correlations have also been found (Takahashi et al., 2010; Papadopoulou et al., 2017). These discrepancies are attributed to different exposure methods during pregnancy, time to initiation of exposure, or duration of exposure. Moreover, we observed no cognitive deficits in prenatal short-term exposure group, which indicates that prenatal insult is associated with exposure duration. To the best of our knowledge, very few studies have investigated the effects of maternal cell phone exposure on older offspring. Furthermore, our study demonstrated that prenatal long-term exposure to the mobile phone increases the risk of cognitive impairment in elderly rat offspring. Thus, indicating that long-term maternal exposure might have life-long effects. Furthermore, our study also showed that EE significantly improved impaired cognition induced by long-term maternal mobile phone exposure in aged offspring. Studies have documented that postnatal EE housing can reverse cognitive deficits caused by prenatal adverse factors such as prenatal stress (Zhang et al., 2012) or ethanol exposure (Wang et al., 2018), however, no evidence exists about the effects of EE on prenatal cell phone exposure. In our study, EE also showed protection against cognitive impairment, but how long the effect of EE will last and when is the best time to initiate the EE treatment remain to be explored.

Previous studies have shown that maternal exposure to mobile phone alters the hippocampal cellular morphology in rodent offspring (Odaci et al., 2008; Bas et al., 2009; Afeefy et al., 2013), though some investigations have demonstrated negative results (Razavinasab et al., 2016). This difference in the studies may be due to a discrepancy in the exposure protocol. In support of this possibility, our results demonstrated altered morphology of the hippocampal cells in elderly rat offspring in prenatal long-term mobile phone exposure group, but not in prenatal short-term exposure group. Moreover, ultrastructural changes in the hippocampal synapses were also observed in prenatal long-term mobile phone exposure group and not in prenatal short-term exposure group. Synapse is an imperative structure in the neurological network, and the fundamental function of a synapse is the transmission of information among neurons. Synaptic density decreases with the aging of the brain (Hedden and Gabrieli, 2004), however, reduced synaptic density was significantly apparent in the prenatal long-term exposure group than the control group, which is beyond healthy aging and indicates the possible occurrence of neurodegeneration. Synaptic loss has been associated with cognitive impairment (Liu et al., 2018). Therefore, it is possible that prenatal long-term mobile phone exposure exacerbates synaptic loss and leads to cognitive deficits in elderly offspring. Also, EE has been reported to increase the number of synapses in the hippocampus after middle cerebral artery occlusion in a mouse model (Wang et al., 2019). Similarly, our study demonstrated that EE rescues hippocampal cells and synaptic changes induced by prenatal long-term mobile phone exposure. This result indicates that EE can counteract the long-term adverse effects of prenatal mobile phone exposure.

Synaptic plasticity characterizes the ability by which synapses can modify the mode of transmission among neurons. SYN is an important protein in presynaptic vesicles, while PSD-95 is a representative member of the PSD protein family (Niethammer et al., 1996). Both SYN and PSD-95 are regarded as important markers of synaptogenesis and play an essential role in synaptic plasticity. In the present research, reduced SYN and PSD-95 expression were observed in the hippocampus of prenatal long-term exposure group but not in the prenatal short-term group, which demonstrates that prenatal long-term phone exposure might lead to a persistent deficit in synaptogenesis and synaptic plasticity in the offspring. Synaptic plasticity is considered to be an underlying mechanism of cognition (Lenck-Santini and Scott, 2015). Animal studies have demonstrated that decreased SYN expression is positively correlated with memory and learning deficits (Schmitt et al., 2009), and the levels of SYN and PSD-95 are reduced in the brains with Alzheimer’s disease (Sze et al., 1997; Kim et al., 2018). Consistent with these previous reports, expression of SYN or PSD-95 was found to be positively correlated with the number of platform crossings in our experiment, which indicates that decreased expression of SYN and PSD-95 may be a potential cause of cognitive impairment. Also, EE housing has been reported to increase the expression of SYN and PSD-95 in the brain, which represents an underlying improvement in synaptic plasticity (Nithianantharajah et al., 2004). Furthermore, EE reverses the reduction in SYN expression induced by exposure to adverse environment during the perinatal period or early life and is regarded as a compensation to the negative effects of adverse perinatal experiences (Koo et al., 2003). In the current study, postnatal EE upregulated SYN and PSD-95 expression in the elderly rat offspring following prenatal long-term mobile phone exposure, implying that EE offers cognitive protection by improving the synaptic plasticity. To our knowledge, limited studies have investigated the effects of EE on synaptic plasticity after prenatal cell mobile phone exposure.

BDNF, a well-studied neurotrophin, is an important facilitator of synaptic plasticity (Gray et al., 2013). Prenatal factors, such as drug or hypoxia-ischemia, have been reported to affect BDNF expression in later life (Griva et al., 2017; Chen et al., 2018). Our research showed that prenatal long-term exposure to a mobile phone but not short-term exposure inhibits BDNF expression in the hippocampus of aged offspring, suggesting that BDNF downregulation is associated with a maternal exposure duration of mobile phone, and prenatal long-term exposure may exert adverse effects until later in the life. Moreover, decreased BDNF expression likely alters synaptic plasticity, ultimately leading to cognitive impairment in an elderly individual following prenatal mobile phone exposure. A previous study has shown that EE increases the level of BDNF protein by modulating BDNF gene expression in various brain disorders, including Huntington’s disease, epilepsy, traumatic brain injury, et cetera (Nithianantharajah and Hannan, 2006). Increased BNDF expression in the brain after exposure to EE can change the neural morphology and synaptic plasticity; moreover, BDNF is the most important neurotrophic factor correlating EE and cognitive improvement (Cao et al., 2014). Moreover, it has already been established that postnatal EE housing can reverse the decrease in BDNF expression induced by prenatal factors such as morphine exposure (Ahmadalipour et al., 2018) or hypoxia-ischemia (Griva et al., 2017). Similarly, our study showed that EE housing after adulthood rescues the reduced BDNF expression caused by maternal long-term exposure, and an increase in the BDNF levels may be the potential reason for improved synaptic plasticity and cognition. Therefore, EE could potentially be an important non-pharmacological therapeutic strategy in the management of cognitive impairment in aged offspring, induced by long-term prenatal mobile phone exposure.

This study was subject to limitations. We only assessed a one-time point among elderly offspring rats; hence, when cognitive impairment occurs in this population remains unclear. Continuous observation at different time points across old age will help to define the duration of the effects of EE. Another limitation is that we did not set up an additional EE control group because the effect of EE on cognition, synaptic plasticity, and BDNF expression has been reported previously (Kempermann et al., 2002; Leal-Galicia et al., 2008). Moreover, our objective was to investigate whether EE counteracts the adverse effect of prenatal mobile phone exposure. Moreover, in our study, the underlying mechanism of EE improving cognitive impairment caused by prenatal mobile phone exposure is not fully elucidated and thus further research is warranted.

In conclusion, the effect of maternal mobile phone exposure on elderly offspring is associated with the duration of exposure. Prenatal long-term mobile phone exposure may aggravate cognitive impairment in the aged rat offspring by modulating the synaptic plasticity and BDNF expression. Moreover, postnatal EE may act as a potential neuroprotective therapeutic strategy by reversing the biochemical changes induced by prenatal long-term mobile phone exposure.

## Data Availability Statement

The datasets generated for this study are available on request to the corresponding author.

## Ethics Statement

The animal study was reviewed and approved by the Ethics Committee of the Second Affiliated Hospital of Fujian Medical University.

## Author Contributions

SH and LW designed the study. MY and HW performed the animal experiments. HH and LW analyzed the experimental results. SH, HH, and LW wrote the first draft of the manuscript. HH contributed equally with SH to this article and could be considered as common first authors. All authors critically read and approved the final manuscript.

## Conflict of Interest

The authors declare that the research was conducted in the absence of any commercial or financial relationships that could be construed as a potential conflict of interest.
